# Self-Test and Self-Calibration of Digital Closed-Loop Accelerometers

**DOI:** 10.3390/s22249933

**Published:** 2022-12-16

**Authors:** Zhiyuan Sun, Miao Wang

**Affiliations:** 1 Key Laboratory of Earthquake Engineering and Engineering Vibration, Institute of Engineering Mechanics, China Earthquake Administration, Harbin 150080, China; 2 Key Laboratory of Earthquake Disaster Mitigation, Ministry of Emergency Management, Harbin 150080, China; 3 Heilongjiang Province Hydraulic Research Institute, Harbin 150080, China

**Keywords:** MEMS accelerometer, digital self-test, digital self-calibration, electromechanical Σ∆

## Abstract

For accelerometers targeted in inertial navigation field, the DC bias error is the most destructive system error, affecting the final precision of long-term dead reckoning. This paper proposes a novel self-test and self-calibration technique for canceling out the DC bias error of the digital closed-loop accelerometers. The self-test of system DC bias is realized by injecting a 1-Bit Σ∆ modulated digital excitation and measuring the second-order harmonic distortion. As illustrated, the second-order harmonic distortion is related to the servo position deviation of the MEMS sensing element, which is one of the main causes of system DC bias error. The automatic capacitance compensation is carried out based on the amplitude and phase information of the detected second-order harmonic distortion, which can dynamically calibrate out the DC bias error. Test results show that there exists a near-linearity relationship between the system DC bias error and the second-order harmonic distortion, which is consistent with the proposed theoretical deduction. Based on the proposed method, the system DC bias error is effectively reduced from 150 to 4 mg, and unaffected by external acceleration bias.

## 1. Introduction

In the past 30 years, important progress has been made in MEMS (Micro-electromechanical Systems) fabricating technology [1]. Benefiting a lot from this, the short-term performance of MEMS accelerometer has shown significant improvement.

However, the MEMS structure inherently suffers from more severe long-term drift problems, compared to traditional mechanical sensing elements [2]. The long-term DC bias drift is the most serious problem among the parameters. This is because, in inertial navigation applications, the DC acceleration bias will be integrated twice to get the position information, and thus it is destructive to the system positioning accuracy [3]. In order to solve the problem, self-test and self-calibration techniques are used to calibrate the long-term DC bias drift out as an effective method [4,5,6,7]. Researchers have put a lot of efforts into this subject in recent years. Some research used the electrostatic force to simulate the physical acceleration to excite the MEMS sensing element and measure the output voltage directly to calculate the drift [8,9]. However, these methods rely on the ideal relationship between electrostatic voltage and the corresponding excitation force, in reality, the relationship itself will suffer from long-term drift problems. Besides that, the accelerometer is sensitive to the earth’s gravity [10,11]. On the earth’s gravitation field, the sensing element is imposed an unknown earth gravity, which depends on the placing status or latitude and altitude. Thus, it imposes an indistinguishable bias error on the sensing element. Simply varying the excitation electrostatic force cannot extract DC bias due to the MEMS drift apart from DC bias caused by the earth’s gravity. These previous methods cannot realize the self-test and self-calibration of DC bias error in the field (they rely on a zero-gravity placing status).

In this paper, we propose a novel self-test and self-calibration method for online testing and calibrating out the DC bias drift of the digital closed-loop MEMS accelerometer. The DC bias drift is detected by a harmonic distortion-based method, which uses the digital 1-Bit Σ-∆ modulated sinusoidal excitation to excite the inner nonlinearity of the closed-loop accelerometer [12]. The closed-loop DC bias drift can be detected by sensing the second-order harmonic distortion, which is proportional to the servo deviation of MEMS proof mass. Further, the capacitance compensation mechanism is established according to the amplitude and phase detection. This mechanism could calibrate out the DC bias drift of the closed-loop accelerometer automatically. The proposed method uses the relationship between nonlinearity and DC bias drift as the calibrating reference, unlike the previous methods, which use the absolute precision of electrostatic force. Furthermore, the nonlinearity property of the digital closed-loop accelerometer is unchanged by external acceleration input due to the servo-feedback characteristics. Thus, the proposed method also has the ability to resist external acceleration affection, which can be carried out online at any placing status.

## 2. System Architecture

**Figure 1:**
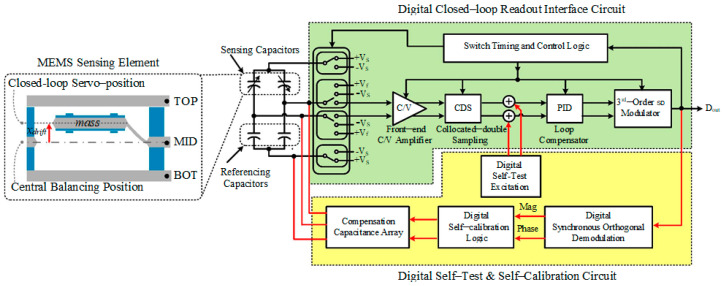
The system architecture of the proposed digital closed-loop readout interface circuit and digital self-test and self-calibration circuit.

In this paper, we propose a method for closed-loop accelerometer online self-test and self-calibration of the DC bias. The system architecture proposed is shown in Figure 1. This system contains a digital closed-loop interface circuit and an auxiliary digital self-test and self-calibration loop. In the digital closed loop, the MEMS sensing element is servo controlled by a digital Σ∆ modulated electrostatic force, which is proportionate to the digital output signal *D_out_*. Since the output is a 1-Bit Σ∆ modulated bit stream, the linearity is guaranteed, and the need for high precision D/A converter is alleviated. Although there is a large amplitude of quantization noise introduced by1-Bit quantizer, it is shaped to high frequency both by the electrical loop filter and the MEMS sensing element. Thus, the in-band noise is kept unaffected, and a digitalized high-performance closed-loop accelerometer is obtained. The working principle of digital Σ∆ closed-loop accelerometer is well illustrated in many other papers, we will not reiterate here and concentrate on the digital self-test and self-calibration loop [13,14,15].

**Figure 2:**
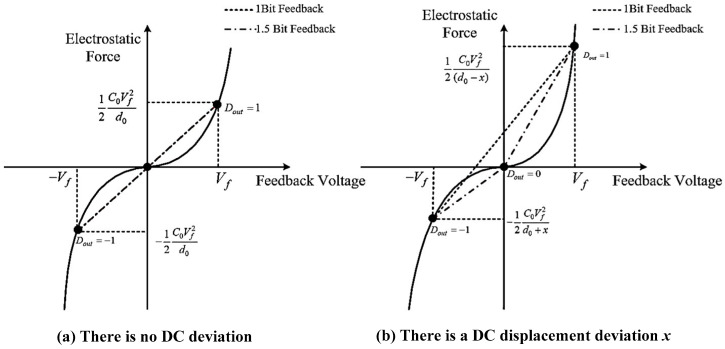
Comparison of the linearization effect between 1-Bit Feedback and 1.5-Bit Feedback.

The digital self-test and self-calibration loop contains the following parts: The digital self-test excitation source, the digital synchronous orthogonal demodulation circuit, the digital self-calibration algorithm logic, and the compensation capacitance array. First of all, the digital self-test excitation source generates a single frequency 1-Bit Σ∆ modulated sinusoidal excitation and injects it into the closed loop readout interface circuit in front of the high-gain section (e.g., the PID loop compensator). The injected self-test signal *V_T_* will inversely transfer from the MEMS sensing element to the system output since there exists high in-band gain provided by the integral section of PID loop compensator. Thus, the inner property of the MEMS sensing element is excited by the digital self-test excitation. Ideally, the MEMS sensing element will be servo-controlled at its balancing position. The square-law relationship could be linearized by using 1-Bit feedback, as shown in Figure 2. The same linearization effect can be obtained by taking either three points in 1.5-Bit feedback or two points in 1-Bit feedback from the square-law relationship of electrostatic force and feedback voltage. However, the 1.5-Bit feedback curve will be a broken line when there is a DC displacement deviation x, whereas 1-Bit force feedback curve will still be a straight line. This is because whenever you take a two-point approximation of whatever curve, the approximation curve will always be a straight line, whereas there are inevitably parasitic and residue stress in the MEMS structure which induces the deviation of the closed-loop servo position to the central balancing position. The DC bias of the closed-loop accelerometer will drift, and the linearity performance is destructed in this circumstance which will be discussed in detail. Thus, the nonlinearity or, in other words, the harmonic distortion is chosen to be a flag to identify the output DC bias drift. An on-chip harmonic distortion analysis unit is realized by the digital synchronous orthogonal demodulation circuit, which could extract the magnitude and phase information of the second-order harmonic distortion in the output signal under self-test mode. According to the magnitude and phase information extracted, a digital self-calibration algorithm is established, which could automatically tune the value and direction of the compensation capacitance array. Therefore, the imbalance of the front-end capacitance bridge is compensated, and the output DC bias is calibrated.

## 3. Harmonic Distortion-Based Self-Test Technique

**Figure 3:**
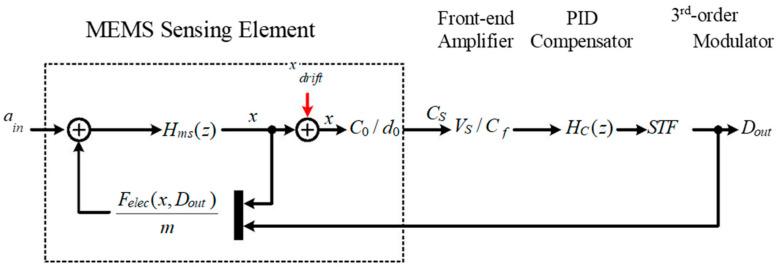
The small signal flow diagram of the whole system.

The proposed self-test technique is based on the relationship between harmonic distortion and output bias drift. In this section, we will give a detailed deduction. The small signal flow diagram of the whole system is shown in Figure 3.

Where, $H_{ms}\left(z\right)$ is the discrete-time transfer function from input acceleration *a_in_* to the displacement variation *x*, *C*_0_ is the static value of sensing capacitance, *d*_0_ is static gap clearance, *V_S_* is the reference voltage applied onto the sensing capacitance at the capacitance detection stage, *C_f_* is the feedback capacitance of capacitance detection circuit, $H_{C}\left(z\right)$ is the transfer function of the PID loop compensator, *STF*_Σ∆_ is the signal transfer function of 3-order electrical Σ∆ modulator. In ideal circumstances, there is no servo deviation, *x_drift_* is equal to zero. The proof mass is located in the middle of fixed plates, and the clearance on either side is equal to *d*_0_. There will be a corresponding displacement variation *x* when there is an input acceleration *a_in_*. This displacement is close to zero when the in-band loop gain is large enough, whereas, when there is a stress or parasitic mismatch in the MEMS sensing element, the proof mass will be servo-controlled at the imbalance position. Thus, there is a deviation *x_drift_* between the closed-loop servo-position and the central balancing position. Under this condition, the DC operating point of the displacement variation will change to $x^{\prime }$, which equals the sum of *x* and *x_drift_* (e.g., $x^{\prime }=x+x_{drift}$).

The electric potential energy between plates of MEMS accelerometer is:

(1) $$U_{C}=\frac{1}{2}QV_{f}=\frac{1}{2}C_{S}V_{f}^{2}$$

The electric force is the gradience of electric potential energy, which can be written as follows:

(2) $$F_{elec}=\frac{1}{2}\frac{C_{0}V_{f}^{2}}{{\left(d_{0}-x\right)}^{2}}$$

The feedback force *F_elec_* and the applied electrostatic voltage *V_f_* represent a square-law relationship. Although using fully differential feedback can linearize it, the residue displacement also contributes to the nonlinearity, especially when there is a DC deviation. The existence of nonlinearity is detrimental to the feedback control system: (1) The linearity of the system will be degraded, and harmonic distortion will be introduced in the output. (2) Out of band quantization noise can be demodulated into the band of interest through this nonlinearity relationship, elevate the in-band noise floor, reduce SNR. (3) If the above problem is severe enough, the stability condition may not be sustained, and the system may be pushed into malfunction.

Since the injection point of self-test excitation is in front of the PID loop compensator, which provides the main in-band gain of the control loop, the transfer function from the self-test excitation *V_T_* to the digital output *D_out_* is mainly determined by the MEMS sensing element and front-end charge amplifier. In other words, the following relationships can be held.

(3) $$\frac{F_{elec}(x,D_{out})}{m}\cdot H_{ms}=x$$

(4) $$H_{ms}\left(z\right)=\frac{T_{fb}}{T_{s}}\frac{T_{s}^{2}z^{-1}}{\left(1-z_{1}z^{-1}\right)\left(1-z_{2}z^{-1}\right)}\cdot \left(\left(1-\frac{T_{d}}{T_{s}}\right)+\frac{T_{d}}{T_{s}}z^{-1}\right)$$

(5) $$x+x_{drift}=V_{T}\cdot \frac{C_{f}d_{0}}{V_{S}C_{0}}$$

*F_elec_*(*x*,*D_out_*) is the expression of digital electrostatic feedback force, which is not only related to the digital output *D_out_* but also to the residue displacement variation *x* when taking the displacement modulation effect into consideration. The relationship can be written as:

(6) $$F_{elec}=\frac{F_{0}}{2}\cdot \left(\frac{1+{\tilde{D}}_{out}}{{\left(1+\delta_{x}\right)}^{2}}-\frac{1-{\tilde{D}}_{out}}{{\left(1-\delta_{x}\right)}^{2}}\right)$$

where, $F_{0}=\frac{1}{2}\frac{C_{0}V_{F}^{2}}{d_{0}}$ is the static coefficient of electrostatic force; $\delta_{x}=\frac{x}{d_{0}}$ is the relative displacement ratio; ${\tilde{D}}_{out}=\frac{1}{N}\overset{N}{\underset{n=1}{\sum }}D_{out}[n]$ is the analog counterpart represented by a digital output.

The following relationship can be obtained by using Equations (4)–(6):

(7) $$\begin{array}{l} \frac{1}{4m}\frac{C_{0}V_{f}^{2}}{d_{0}^{2}}\cdot (\frac{1+{\tilde{D}}_{out}}{(1-\frac{x_{drift}}{d_{0}}+V_{T}\cdot \frac{C_{f}}{V_{S}C_{0}}{)}^{2}}-\frac{1-{\tilde{D}}_{out}}{(1-\frac{x_{drift}}{d_{0}}-V_{T}\cdot \frac{C_{f}}{V_{S}C_{0}}{)}^{2}})\\ =V_{T}\cdot \frac{C_{f}d_{0}}{V_{S}C_{0}}-x_{drift} \end{array}$$

${\tilde{D}}_{out}$ is the polynomial expansion of the self-test harmonics excitation *V_T_*:

(8) $${\tilde{D}}_{out}=\eta_{0}\cdot V_{T}^{0}+\eta_{1}\cdot V_{T}^{1}+\eta_{2}\cdot V_{T}^{2}+\eta_{3}\cdot V_{T}^{3}+\ldots$$

The harmonic distortion coefficients *η_i_* can be obtained by substituting Equation (8) into Equation (7) and comparing the coefficients of corresponding terms $V_{T}^{i}$.

(9) $$\left[\begin{array}{l} \eta_{0}\\ \eta_{1}\\ \eta_{2}\\ \eta_{3} \end{array}\right]=\left[\begin{array}{l} -\frac{C_{0}V_{f}^{2}/d_{0}^{2}\cdot H_{ms}+d_{0}m}{0.5C_{0}V_{f}^{2}/d_{0}^{2}\cdot H_{ms}}\cdot \frac{C_{f}}{C_{0}}\cdot \frac{x_{drift}}{d_{0}}\\ \frac{C_{0}V_{f}^{2}/d_{0}^{2}\cdot H_{ms}+d_{0}m}{0.5C_{0}V_{f}^{2}/d_{0}^{2}\cdot H_{ms}}\cdot \frac{C_{f}}{C_{0}}\cdot \frac{1}{V_{S}}\\ \frac{3C_{0}V_{f}^{2}/d_{0}^{2}\cdot H_{ms}+9d_{0}m}{0.5C_{0}V_{f}^{2}/d_{0}^{2}\cdot H_{ms}}\cdot \frac{C_{f}}{C_{0}}\cdot \frac{1}{V_{S}^{2}}\cdot \frac{x_{drift}}{d_{0}}\\ -\frac{C_{0}V_{f}^{2}/d_{0}^{2}\cdot H_{ms}+3d_{0}m}{0.5C_{0}V_{f}^{2}/d_{0}^{2}\cdot H_{ms}}\cdot \frac{C_{f}}{C_{0}}\cdot \frac{1}{V_{S}^{3}} \end{array}\right]$$

From Equation (9), we can notice the following things when there is the servo-deviation of MEMS sensing element *x_drift_* ≠ 0:

(1) The even-order harmonic distortion terms will occur in the self-test response.
(2) There is a near-linear relationship between the amplitude of even order harmonic distortion and the servo deviation *x_drift_*.

The above discussion is based on the assumption that *x_drift_* << *d*_0_. However, in the actual sensitive structure of the accelerometer, the conversion from displacement to capacitance is nonlinear, and the drift position cannot fully satisfy the condition, which will introduce nonlinearity part in the second-order harmonic distortion. Inevitably, parasitic mismatch capacitance is introduced by routing, bonding wire, and packaging. When the MEMS sensing element is incorporated in a closed servo loop, the mismatch in sensing capacitance will cause the proof mass to be servo-controlled in the wrong position, and there is a displacement deviation *x_drift_*. As shown in Equation (6), it will introduce a DC bias term η and contribute to the final output DC bias.

## 4. Digital Automatic Self-Calibration

**Figure 4:**
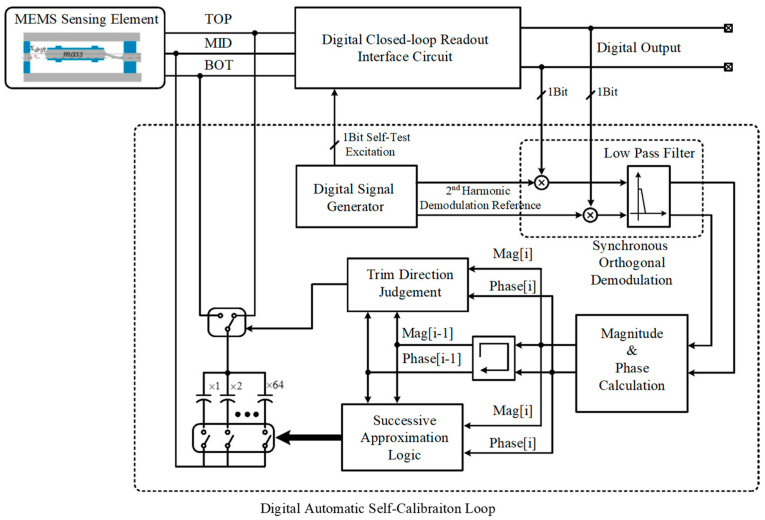
The diagram of the digital automatic self-calibration circuit.

Based on the above theory, this section proposes an automatically on-chip digital self-calibration circuit. The diagram of the proposed self-calibration circuit is shown in Figure 4.

Firstly, the MEMS accelerator is excited to oscillate by the digital self-test excitation. The self-test excitation is also a 1-Bit Σ∆ modulated to avoid introducing additional distortion. Thus, the linearity of the excitation source is inherently guaranteed by the 1-Bit excitation. The excitation has the flowing form:

(10) $$V_{T}(t)=A_{T}\cos(\omega_{T}t)+Q_{n}(t)$$

where *Q_n_*(*t*) is the quantization noise introduced by the 1-Bit Σ∆ modulation. The second-order harmonic distortion is extracted from the output bitstream of the digital Σ∆ closed-loop accelerometer. The synchronous orthogonal demodulation is used in order to suppress the accompanying quantization noise in the output bitstream. Besides, it also extracts the phase information of the detected second-order harmonic so as to determine the calibrating direction.

The orthogonal demodulation signal is generated from the same digital signal source with the self-test excitation. Since these two signals use the same reference clock, the precision frequency and phase relationship are guaranteed. The referencing demodulation signal is a 24-Bit sinusoidal signal generated by a look-up-table, which can be expressed as:

(11) $$V_{T}(t)=A_{r}\cos(2\omega_{T}t+\varphi )$$

Since the output signal of the Σ∆ closed-loop accelerometer is a single-bit one, the 24-Bit × 1-Bit multiplier can be greatly simplified by a multiplexer. After the demodulation, the output signal can be expressed:

(12) $$\begin{array}{l} Y(t)=A_{r}\cos(2\omega_{T}+\varphi )\times \left(\overset{\infty }{\underset{n=0}{\sum }}U_{n}\cos(n\omega_{T}t)+n(t)\right)\\ =\frac{A_{r}U_{2}}{2}\cos(\varphi )+\overset{\infty }{\underset{n=1}{\sum }}\frac{A_{r}U_{n}^{\prime }}{2}\cos(n\omega_{T}t+\varphi )+A_{r}\cos(2\omega_{T}+\varphi )n(t) \end{array}$$

The output *Y*(*t*) contains the following tips after demodulation according to Equation (12):

(1) ADC component contains the magnitude and phase information of the second-order harmonic of self-test response.
(2) The other harmonic distortion component is modulated to *nω_T_* and will be filtered out by a succeeding low-pass filter.
(3) Since the noise varies randomly, only the component which has both the same frequency and phase relationship with the second-order harmonic is demodulated to baseband. Thus, most of the noise gets suppressed.

In order to extract the magnitude and phase of the second-order harmonic, orthogonal demodulation is used in this design. Two orthogonal sinusoidal demodulation signals are generated by the digital signal source. The in-phase and quadrature-phase output is denoted *Y_I_* and *Y_Q_*, respectively, which can be expressed as:

(13) $$\left\{ \begin{array}{l} Y_{Q}=\frac{A_{r}U_{2}}{2}\cos(\varphi )\\ Y_{I}=\frac{A_{r}U_{2}}{2}\sin(\varphi ) \end{array}\right.$$

The magnitude *U* can be calculated by:

(14) $$U=\sqrt{Y_{I}^{2}+Y_{Q}^{2}}=\frac{A_{r}U_{2}}{2}$$

The phase angle φ can be calculated by:

(15) $$\varphi =\arctan(\frac{Y_{I}}{Y_{Q}})$$

According to the phase and magnitude information extracted, an auto-calibrating mechanism is established. The basic idea is a successive approximating trial-and-error procedure. This procedure starts from the MSB (Most Significant Bit) of the binary-weighted compensation capacitance, whose value is 2*^N^* × ∆*C*. In each step, the current control bit is set to ‘1’ firstly, and the capacitance with the value 2^*i*^ × ∆*C* is set to parallel with the sensing capacitance.

Each control bit is determined in the direction of reducing the self-test harmonic distortion. Thus, in the end of the calibration procedure, the final sensing capacitance mismatch will be trimmed in the range of −∆*C* ∼ +∆*C*.

## 5. Test Results and Discussion

**Figure 5:**
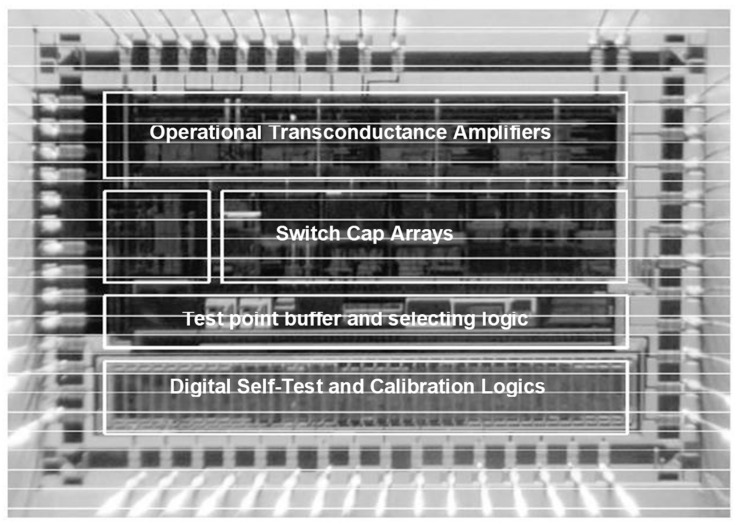
The micro-photograph of the die of the ASIC.

**Figure 6:**
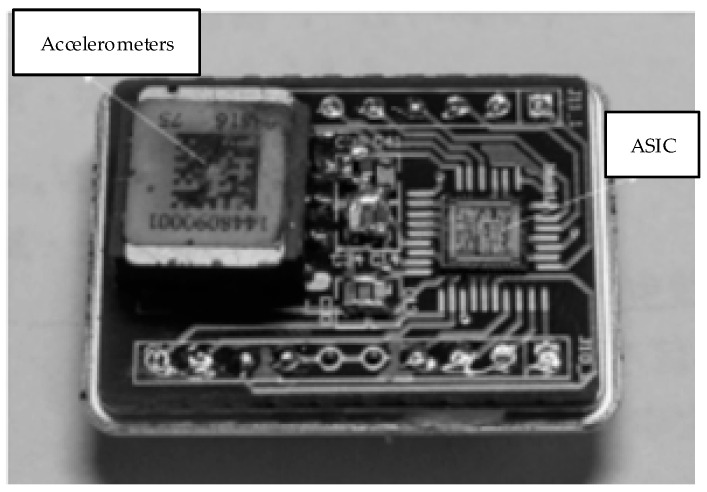
The picture of fabricated MEMS accelerometer and the packaged device.

A prototype readout ASIC (Application Specific Integrated Circuit) with proposed self-test and self-calibration function was designed and fabricated in 0.35 µm CMOS-BCD process with a chip area of 4000 × 3800 µm. Figure 5 is the micro-photograph of the die of the proposed ASIC. The chip is supplied with a ±2.5 V voltage and uses a 12 V high voltage electrostatic feedback to generate the servo force. The clocking frequency of the whole chip is 500 kHz. The total power consumption is 35 mW. The picture of the fabricated MEMS accelerometer and the packaged device under the test is shown in Figure 6.

**Figure 7:**
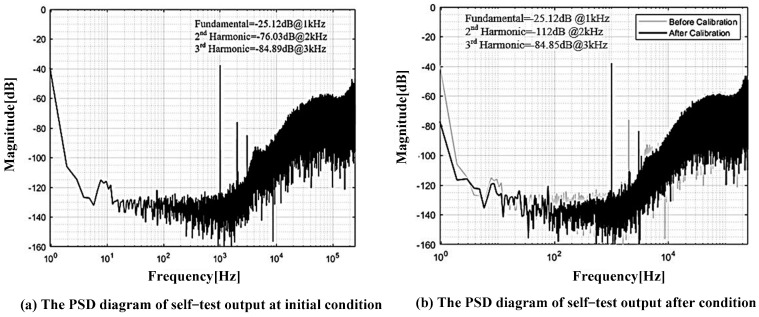
The power spectrum density diagram of the output bitstream.

The parameters of MEMS accelerometer are listed in Table 1. The sensor is first placed at the 0 g condition, and the self-test excitation is injected to verify the self-test function and the proposed bias drift theory based on harmonic distortion. The injected excitation is a 1-Bit Σ∆ modulated digital sinusoidal with a frequency of 1 kHz and the equivalent amplitude of 10 mg. The responding digital output of the accelerometer is captured by a logic analyzer, and FFT analysis is performed to analyze the harmonic distortion. Figure 7 presents the PSD (power spectrum density) diagram of the digital closed-loop accelerometer’s output under the self-test excitation. Figure 7a presents the PSD diagram before calibration. From the above analysis, we can find that the MEMS sensing element is not servo-controlled at the central balancing position under the initial condition, and there exists an obvious second-order harmonic distortion with an amplitude of −76 dB and a DC bias with an amplitude of −40 dB. After calibration, both the harmonic distortion and the output DC bias are reduced, as shown in Figure 7b. The second-order harmonic distortion is reduced to −112 dB, and the output DC bias is reduced to −78 dB. These results show that it is feasible to use the harmonic distortion as the flag to calibrate the output bias drift of the digital closed-loop accelerometer.

**Figure 8:**
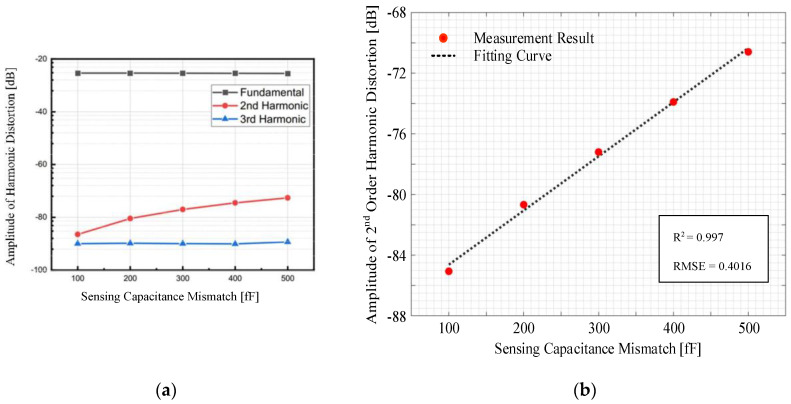
The harmonic distortion with respect to the mismatch in sensing capacitances. (**a**) Amplitude of harmonic distortions with different sensing capacitance mismatch. (**b**) Measurement RMSE results for the second-order harmonic distortion.

Next, the front-end sensing capacitance mismatch is manually tuned, and the self-test procedure is repeated. The obtained amplitude of each harmonic distortion versus the capacitance mismatch pre-imposed is shown in Figure 8. It can be found that the second-order harmonic distortion approximately increases linearly with the capacitance mismatch pre-imposed at the measurement RMSE of 0.4016 and R^2^ of 0.997, whereas the third-order harmonic distortion is unaffected. There exists a nonlinearity part in the relationship between second-order harmonic distortion and mismatch of sensing capacitance which is caused by non-ideal drift and displacement.

**Figure 9:**
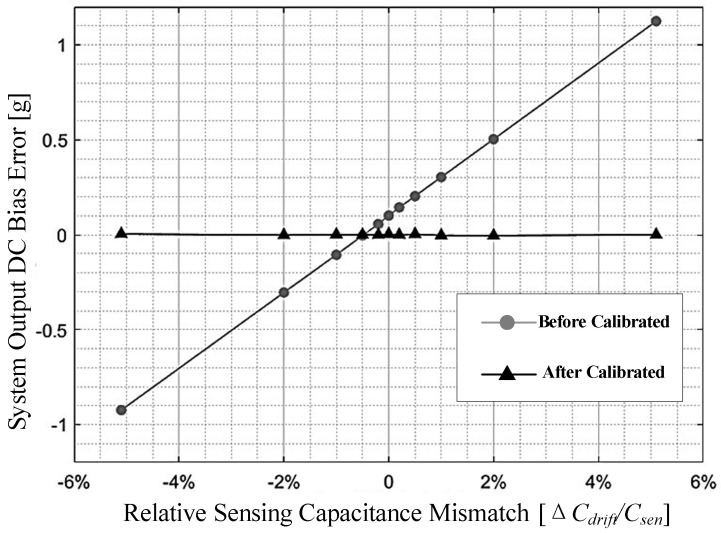
System output DC bias error with respect to the mismatch in sensing capacitances.

Under the different capacitance mismatch conditions, the self-calibration is taken on. The output DC bias error of the digital closed-loop accelerometer before and after self-calibration is shown in Figure 9. As shown, the front-end sensing capacitance mismatch will directly induce output DC error. The relative capacitance mismatch from −5% to +5% will translate to a DC bias error −1 ∼ +1 g. After calibration, the capacitance mismatch is automatically compensated by using a calibration capacitance array. The output DC bias error is calibrated into the range of −4 ∼ +4 mg.

**Figure 10:**
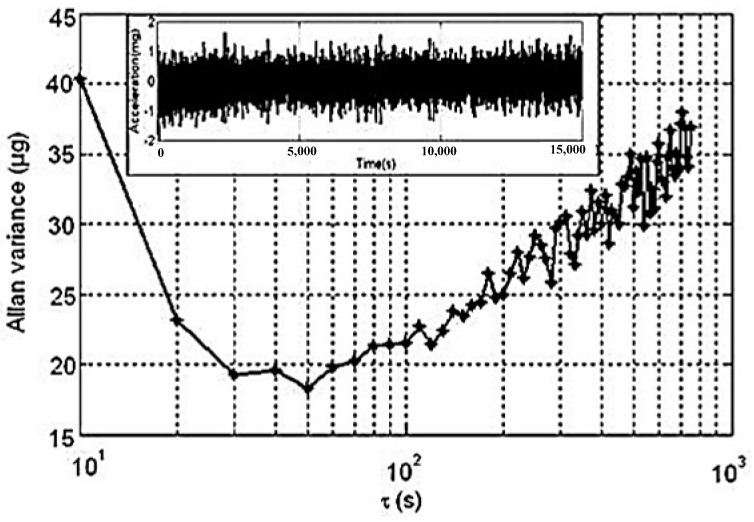
Allan deviation plot of accelerometer.

Bias instability performance is tested at room temperature, and the output of the digital closed-loop MEMS accelerometer is recorded for about 4 h, as shown in Figure 10. The bias instability after the calibration is about 18 μg.

## 6. Conclusions

In this paper, we proposed an on-chip self-test and self-calibration method for the DC bias error of a digital closed-loop MEMS accelerometer. The method is based on the relationship between harmonic distortion and DC bias error. It has been proven in theory and experiments that the self-test response will exhibit even-order harmonic when there is a servo-position deviation induced by drift. The amplitude of even-order harmonic is linearly related to the output DC bias error. On this foundation, a successive approximation self-calibration circuit is established. It uses the second-order self-test harmonic distortion as a flag and automatically calibrates out the front-end sensing capacitance mismatch. After calibration, the DC bias error could be effectively reduced to the range of −4 ∼ +4 mg.

## Figures and Tables

**Table 1:** Properties summary and comparison.

Physical Quality	Value
Sensitivity	10 pF/g
Quality	62 × 10^−6^ kg
Static capacitance	180 pF
Coefficient of damping	0.01 N/m/s
Spacing of comb	2 μm
Coefficient of stiffness	2760 N/m
Resonant frequency	1000 Hz
Quality Factor	>30
Brown Noise Equivalent Acceleration	<60 ng/Hz^1/2^

## Data Availability

Not applicable.

## References

[1] Shkel A.M. Precision navigation and timing enabled by microtechnology: Are we there yet?. Proceedings of the ION 2013 Pacific PNT Meeting.

[2] Deb N., Blanton R.D. Built-in self test of CMOS-MEMS accelerometers. Proceedings of the International Test Conference.

[3] Trusov A.A., Zotov S.A., Simon B.R., Shkel A.M. Silicon accelerometer with differential frequency modulation and continuous self-calibration. Proceedings of the 2013 IEEE 26th International Conference on Micro Electro Mechanical Systems (MEMS).

[4] Balachandran G.K., Petkov V.P., Mayer T., Baislink T. 27.1 a 3-axis gyroscope for electronic stability control with continuous self-test. Proceedings of the 2015 IEEE International Solid-State Circuits Conference—(ISSCC) Digest of Technical Papers.

[5] Basith I.I., Kandalaft N., Rashidzadeh R. Built-in self-test for capacitive MEMS using a charge control technique. Proceedings of the 2010 19th IEEE Asian Test Symposium.

[6] Dhayni A., Mir S., Rufer L. MEMS built-in-self-test using MLS. Proceedings of the Ninth IEEE European Test Symposium, ETS 2004.

[7] Xiong X., Wu Y., Jone W. (2005). A dual-mode built-in self-test technique for capacitive MEMS devices. IEEE Trans. Instrum. Meas..

[8] Chu Y., Liu Y., Dong J., Chi B. Elimination of nonlinearity in Σ∆ MEMS accelerometer. Proceedings of the 2015 IEEE SENSORS (2015).

[9] de Bruin D., Allen H., Terry S. Second-order effects in self-testable accelerometers. Proceedings of the IEEE 4th Technical Digest on Solid-State Sensor and Actuator Workshop (1990).

[10] Frosio I., Stuani S., Borghese N.A. Auto calibration of MEMS accelerometer. Proceedings of the 2006 IEEE Instrumentation and Measurement Technology Conference.

[11] Yu H., Ye L., Guo Y., Su S. (2020). An Innovative 9-Parameter Magnetic Calibration Method Using Local Magnetic Inclination and Calibrated Acceleration Value. IEEE Sens. J..

[12] Painter C., Shkel A. (2003). Active structural error suppression in MEMS vibratory rate integrating gyroscopes. IEEE Sens. J..

[13] Petkov V., Boser B. (2005). A fourth-order/spl Sigma//spl Delta/interface for micromachined inertial sensors. IEEE J. Solid-State Circuits.

[14] Ismail A.H., Elsayed A. Σ − ∆ based force-feedback capacitive micro-machined sensors: Extending the input signal range. Proceedings of the 2017 IFIP/IEEE International Conference on Very Large Scale Integration (VLSI-SoC).

[15] Grinberg B., Feingold A., Koenigsberg L., Furman L. Closed-loop MEMS accelerometer: From design to production. Proceedings of the 2016 DGON Intertial Sensors and Systems(ISS).

